# A Mitochondrial Superoxide Signal Triggers Increased Longevity in *Caenorhabditis elegans*

**DOI:** 10.1371/journal.pbio.1000556

**Published:** 2010-12-07

**Authors:** Wen Yang, Siegfried Hekimi

**Affiliations:** Department of Biology, McGill University, Montreal, Quebec, Canada; University of Massachusetts Medical School, United States of America

## Abstract

The *nuo-6* and *isp-1* genes of *C. elegans* encode, respectively, subunits of complex I and III of the mitochondrial respiratory chain. Partial loss-of-function mutations in these genes decrease electron transport and greatly increase the longevity of *C. elegans* by a mechanism that is distinct from that induced by reducing their level of expression by RNAi. Electron transport is a major source of the superoxide anion (O^⋅^^–^), which in turn generates several types of toxic reactive oxygen species (ROS), and aging is accompanied by increased oxidative stress, which is an imbalance between the generation and detoxification of ROS. These observations have suggested that the longevity of such mitochondrial mutants might result from a reduction in ROS generation, which would be consistent with the mitochondrial oxidative stress theory of aging. It is difficult to measure ROS directly in living animals, and this has held back progress in determining their function in aging. Here we have adapted a technique of flow cytometry to directly measure ROS levels in isolated mitochondria to show that the generation of superoxide is elevated in the *nuo-6* and *isp-1* mitochondrial mutants, although overall ROS levels are not, and oxidative stress is low. Furthermore, we show that this elevation is necessary and sufficient to increase longevity, as it is abolished by the antioxidants NAC and vitamin C, and phenocopied by mild treatment with the prooxidant paraquat. Furthermore, the absence of effect of NAC and the additivity of the effect of paraquat on a variety of long- and short-lived mutants suggest that the pathway triggered by mitochondrial superoxide is distinct from previously studied mechanisms, including insulin signaling, dietary restriction, ubiquinone deficiency, the hypoxic response, and hormesis. These findings are not consistent with the mitochondrial oxidative stress theory of aging. Instead they show that increased superoxide generation acts as a signal in young mutant animals to trigger changes of gene expression that prevent or attenuate the effects of subsequent aging. We propose that superoxide is generated as a protective signal in response to molecular damage sustained during wild-type aging as well. This model provides a new explanation for the well-documented correlation between ROS and the aged phenotype as a gradual increase of molecular damage during aging would trigger a gradually stronger ROS response.

## Abstract 2

The study of long-lived *C. elegans* mutants suggests that mitochondrial oxidants can actually help reduce aging by acting as stress signals, rather than acting solely as toxic molecules.

## Author Summary

An unequivocal demonstration that mitochondria are important for lifespan comes from studies with the nematode *Caenorhabditis elegans.* Mutations in mitochondrial proteins such as ISP-1 and NUO-6, which function directly in mitochondrial electron transport, lead to a dramatic increase in the lifespan of this organism. One theory proposes that toxicity of mitochondrial reactive oxygen species (ROS) is the cause of aging and predicts that the generation of the ROS superoxide should be low in these mutants. Here we have measured superoxide generation in these mutants and found that it is in fact elevated, rather than reduced. Furthermore, we found that this elevation is necessary and sufficient for longevity, as it is abolished by antioxidants and induced by mild treatment with oxidants. This suggests that superoxide can act as a signal triggering cellular changes that attenuate the effects of aging. This idea suggests a new model for the well-documented correlation between ROS and the aged phenotype. We propose that a gradual increase of molecular damage during aging triggers a concurrent, gradually intensifying, protective superoxide response.

## Introduction

Mitochondrial function has been linked to the aging process in a number of ways [1]. In particular, mitochondria are crucial in energy metabolism and as such have been implicated in the aging process by one of the very first theories of aging [2], the rate-of-living theory of aging [3], which suggested that the rate of aging is proportional to the rate of energy metabolism (reviewed in [4]). Mitochondrial function in animals is also known to decline with age [5],[6], which, together with the finding that mitochondria are an important source of toxic reactive oxygen species (ROS), has led to the oxidative stress (or free radical) theory of aging [7],[8].

Two types of mutations that affect mitochondrial function have been found to affect the rate of aging in *C. elegans*, mutations that shorten lifespan, such as *mev-1* [9] and *gas-1* [10], and mutations that lengthen lifespan, such as *clk-1* [11], *isp-1* [12], *lrs-2* [13], and *nuo-6* [14]. *lrs-2* encodes a mitochondrial leucyl-tRNA-synthetase, and its effect on the function of mitochondrial electron transport is likely relatively indirect, via partial impairment of mitochondrial translation. However, *clk-1* encodes an enzyme necessary for the biosynthesis of ubiquinone, a lipid antioxidant and an electron transporter of the respiratory chain [15], and m*ev-1*, *gas-1*, *isp-1*, and *nuo-6* all encode subunits of mitochondrial respiratory complexes. On the strength of the oxidative stress theory of aging it has been suggested, and supported by a number of observations (reviewed in [16],[17]), that the *mev-1* and *gas-1* mutations reduce lifespan by increasing mitochondrial oxidative stress, and *clk-1*, *isp-1*, and *nuo-6* increase lifespan by reducing it.

In addition to genomic mutations that affect mitochondrial proteins, it has been found that knockdown by RNA interference of *C. elegans* genes that encode subunits of mitochondrial complexes, including *isp-1* and *nuo-6*, also prolongs lifespan [13],[18],[19]. Although the effect of RNAi on ETC subunits, which is conserved in *Drosophila* [20], was initially believed to be similar to that of the mutations [21],[22],[23], it was recently found that it is in fact distinct and separable [14].

A recent study analyzed patterns of gene expression in *isp-1* mutants together with those in *clk-1* and *cyc-1(RNAi)* [23] and suggested that the overlap between these patterns could define the biochemical processes that underlie the effect of all interventions that impact mitochondria. However, our recent findings that *isp-1(qm150)* and *isp-1(RNAi)* trigger fully separable mechanisms suggests that the overlapping gene expression changes identified by Cristina et al. [23] might not be sufficient to prolong lifespan. Rather some of the gene expression changes that are specific to each type of intervention are necessary for their effect on lifespan and can act additively.

*isp-1* mutants show a trend toward low levels of oxidative damage to proteins, increased expression of the cytoplasmic Cu/Zn superoxide dismutase (SOD-1) and of the mitochondrial Mn superoxide dismutase (SOD-2) [24], and increased resistance to acute treatment with the prooxidant paraquat [14]. However, although knocking down the genes encoding the major superoxide dismutase by RNAi results in normal or elevated levels of oxidative damage, it had no effect on the lifespan of the mutants [24], suggesting that the reduced oxidative damage found in *isp-1* mutants is not responsible for their longevity. Furthermore, the notion that mitochondrial oxidative stress could be the cause of aging has recently been challenged by a number of studies in *C. elegans* [24],[25],[26],[27],[28], in *Drosophila* [29], and in mice (reviewed in [30]).

ROS are not just toxic metabolites that lead to oxidative stress but are also signaling molecules that are believed to be involved in a mitochondria-to-nucleus signaling pathway that could impact aging [1],[31],[32],[33]. Interfering with mitochondrial function has the potential to alter the rate and/or the pattern of production of ROS by mitochondria, including in counter-intuitive ways. For example, reducing oxygen concentration increases ROS production by mitochondrial complex III in vertebrate cells [34],[35], and the knockout of *sod-2* in *C. elegans* can lead to normal [25] or increased lifespan in spite of increased oxidative damage [26]. Here we examined ROS production by mitochondrial mutants and found that *isp-1* and *nuo-6* mutants have increased generation of the superoxide anion but not increased levels of other ROS and that this increase is necessary and sufficient for longevity, suggesting that superoxide triggers mechanisms that slow down aging, presumably at the level of gene expression.

## Results

### *isp-1*, *nuo-6*, and *daf-2* Mutant Mitochondria Display Elevated Generation of Superoxide But Not of Overall ROS

**Figure 1:**
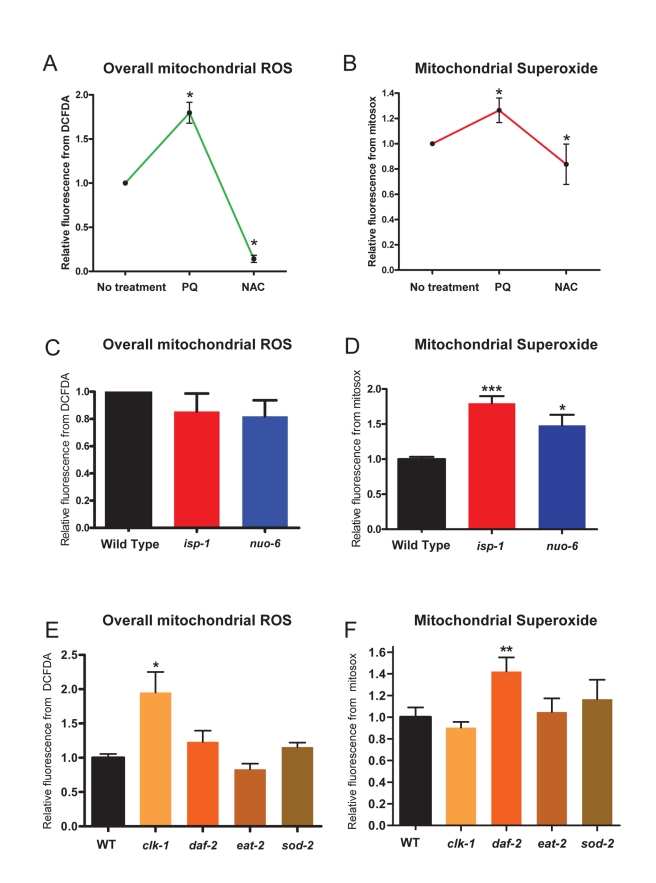
Reactive oxygen species (ROS) in isolated mitochondria of long-lived mutants and in response to paraquat (PQ) and N-acetyl-cysteine (NAC) treatment. Global ROS levels were measured by quantifying the fluorescence of the reporter dye H_2_DCFDA, and superoxide with the dye MitoSox, in FACS-sorted mitochondria. Values are normalized to the value of the untreated sample or the wild type. PQ and NAC, respectively, increase and decrease the levels of both global ROS (A) and superoxide (B). Mitochondria isolated from *isp-1(qm150)* and *nuo-6(qm200)* mutants show slightly decreased global ROS generation (C) but significantly increased superoxide generation (D). Mitochondria from *clk-1* mutants, but not from *daf-2(e1370)*, *eat-2(ad1116)*, and *sod-2(ok1030)* mutants, show significantly increased global ROS levels (E). Mitochondria from *daf-2* mutants, but not from *clk-1*, *eat-2*, and *sod-2* mutants, show increased superoxide levels (F). * *p*<0.05, ** *p*<0.001, *** *p*<0.0001, by the paired *t* test.

To measure changes in mitochondrial ROS generation that could affect signaling, it is not adequate to measure the level of ROS damage, as a change in ROS damage levels can be brought about by changes in detoxification of ROS, in protein turnover, or in damage repair. However, it is notoriously difficult to directly visualize or measure ROS generation and ROS levels in intact organisms including in living worms. To overcome this difficulty we have adapted a technique originally developed for vertebrates that uses flow cytometry to sort isolated intact mitochondria and measure ROS levels with indicator dyes (Figure S1) [37]. Mitochondria were extracted from worms by standard techniques and loaded with either one of two fluorescent indicator dyes, H_2_DCFDA, a dye that is sensitive to a variety of ROS but rather insensitive to superoxide [38],[39], and MitoSox, a dye that is exclusively sensitive to superoxide [40]. The prooxidant paraquat (PQ) induces mitochondrial superoxide generation [41], and the antioxidant N-acetyl-cysteine (NAC) has an antioxidant effect on all types of ROS [42],[43]. As expected, when purified mitochondria were treated with PQ, the fluorescence of both H_2_DCFDA and MitoSox increased, and the fluorescence of both decreased when treated with NAC (Figures 1A, 1B, and S1B). One limitation of this technique is the need for a rather large amount of mitochondria. For example, a sufficient amount of worms is not readily obtained from worms treated by RNAi, and we have therefore focused on long-lived mutants only.

We used the cytometry technique to determine the generation of mitochondrial superoxide and of overall mitochondrial ROS in a number of long-lived mutants. Both *isp-1* and *nuo-6* mutations did not affect H_2_DCFDA fluorescence (overall ROS) significantly, but both showed elevated MitoSox fluorescence (superoxide) (Figure 1C and 1D). Mutants of four other genes (*clk-1(qm30)*, *eat-2(ad1116), daf-2(e1370)*, and *sod-2(ok1030)*) were also tested (Figure 1E and 1F). *clk-1* mutants showed an elevation of overall ROS-associated fluorescence but not of superoxide-associated fluorescence. *daf-2* mutants were most similar to the mitochondrial respiratory chain mutants with an elevation of superoxide-associated fluorescence but no significant elevation in overall ROS-associated fluorescence. Finally, *eat-2* and *sod-2* mutants showed no significant elevation in either signal but only a trend for low overall ROS in the case of *eat-2* mutants and a trend for increased superoxide in the case of *sod-2* mutants.

The elevated MitoSox signal in *isp-1*, *nuo-6*, and *daf-2* corresponds mostly to increased superoxide generation, as all three mutants are known for elevated levels of the mitochondrial SOD-2 and SOD-3 [12],[14],[24],[44], whose activity would prevent the accumulation of superoxide. Elevated superoxide detoxification, however, should not prevent measuring increased superoxide generation as superoxide is generated at prosthetic electron carriers such as ubiquinone in complex III [45],[46] and FMN in complex I [47],[48], which are at least partially buried in the complexes. Thus a small molecular weight dye that has access to these sites can trap the superoxide before it has the opportunity to diffuse toward the SOD-2 and SOD-3 proteins. There is no increase in the H_2_DCFDA signal in these mutants likely because this dye is not particularly sensitive to superoxide [49]. It appears therefore that in the presence of efficient detoxification the level of overall ROS is not significantly increased by the increased superoxide generation that we observe. This is consistent with the finding that these mutants do not have increased oxidative damage [14],[24].

*sod-2* deletion mutants do not show a significant increase in the MitoSox signal (Figure 1E and 1F), indicating that decreased detoxification does not lead to an easily measurable increase in this signal in purified mitochondria. The signal from H_2_DCFDA, a dye which has very broad sensitivity but is not very sensitive to superoxide [49], is also unchanged, suggesting that, at least in isolated worm mitochondria, electron transport is not the main source of the type of ROS to which H_2_DCFDA dye is significantly sensitive. The level of superoxide generation in these mutants might also be kept moderately low because of their reduced electron transport [26], although low electron transport could in principle also result in elevated superoxide as we have observed in *isp-1* and *nuo-6* mutants.

*clk-1* mutants have only a small deficit in electron transport [24],[50],[51], in spite of a strongly altered content in quinones [51],[52],[53],[54]. Indeed, while wild-type animals contain endogenously synthesized UQ_9_ as well as a small amount of dietary bacterial UQ_8_, *clk-1* mutants contain only the dietary ubiquinone and no UQ_9_. Here we found that *clk-1* mutants have normal superoxide generation but enhanced overall ROS levels, which suggests that the antioxidant function of UQ_9_ is a crucial sink for mitochondrial ROS, whose absence appears to lead to an increase of overall ROS even in the absence of increase superoxide generation. *eat-2* mutants are long-lived because of reduced food intake (dietary restriction) [55]. Although dietary restriction has been found to impinge on mitochondrial function in other systems, no changes in mitochondrial superoxide and overall ROS signals were observed.

### Elevated Superoxide Promotes Longevity

**Figure 2:**
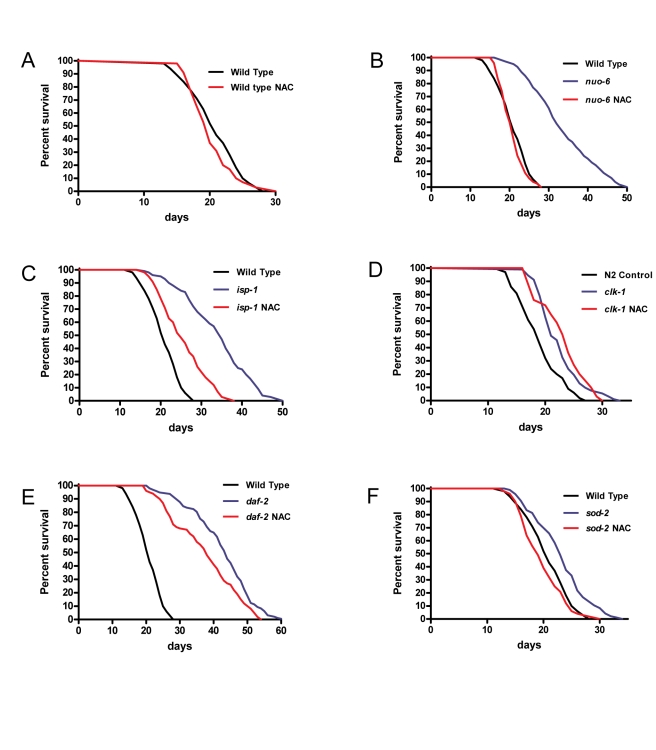
Lifespans of wild-type animals and mutants treated with 10 mM NAC. The treatment has no effect on wild type (A) but dramatically suppresses the lifespan extension of the respiratory chain subunit mutants *nuo-6* (B) and *isp-1* (C). In contrast, it has no lifespan-shortening effects on the long-lived *clk-1* mutants (D). NAC treatment has only a moderate effect on the very long-lived *daf-2* mutants (E) but also completely abolished the extended longevity of *sod-2* mutants (F). See Tables 1 and S1 for details of genotype, sample size, and statistical analysis.

To determine how the elevated superoxide affects the lifespan of mutants, we treated worms with 10 mM of NAC and scored their survival (Figure 2 and Table 1). The treatment had no effect on the survival of the wild type (Figure 2A), which shows that it is not toxic for lifespan at the concentration used. However, NAC treatment fully abolished the increased longevity of *nuo-6* and severely limited that of *isp-1* (Figure 2B and 2C). The lesser effect on *isp-1* is consistent with the larger increase of superoxide in these mutants (Figure 1D), given that the effect of NAC is gradual (1 mM has less effect than 8 mM, which has less than 10 mM; Table S1). At high concentration (>10–15 mM) NAC can be deleterious even on the wild type, but at the concentration used (10 mM) NAC had no effect on the apparent health of the mutants, whose overall aspect after treatment was indistinguishable from that of the untreated worms (Figure S2A). We have also quantified several phenotypes, including defecation, swimming, brood size, and post-embryonic development, after NAC treatment of the wild type and of *nuo-6*, which is the mutant that is most sensitive to NAC (10 mM NAC completely abolishes its increased longevity). Treatment with 1 mM vitamin C also significantly shortened the lifespan of both *isp-1* and *nuo-6* mutants without affecting the wild type (Table S1). Most effects of NAC were quite small (Figure S2B–E), except on the post-embryonic development of the wild type (Figure S2C). Furthermore, for defecation, brood size, and post-embryonic development, the effect of NAC on the mutant produced a change in the same direction as on the wild type but of a lesser extent. Only for swimming is the effect greater on the mutant. But the effect consists of swimming faster after NAC treatment and thus bringing the mutant phenotype closer to the wild-type. We conclude that there is little evidence of an indirect deleterious effect of NAC.

**Table 1:** Longevity after paraquat and NAC treatment.

	Control		PQ			NAC		
	Mean ± SD Sample Size	Maximum Lifespan	Mean ± SD Sample Size	Maximum Lifespan	% Change *p* Value	Mean ± SD Sample Size	Maximum Lifespan	% Change *p* Value
Wild Type (N2)	18.4±3.5	29	29.0±5.1	43	+58%	20.2±3.2	30	+10%
	(*n* = 400)		(*n* = 200)		*p*<0.0001	(*n* = 100)		0.1655
*nuo-6(qm200)*	33.4±7.6	49	36.1±8.3	54	+8%	20.7±3.0	28	−38%
	(*n* = 200)		(*n* = 200)		*p* = 0.0125	(*n* = 73)		<0.0001
*isp-1(qm150)*	33.9±7.9	53	35.0±7.3	50	+3%	25.6±5.6	38	−24%
	(*n* = 200)		(*n* = 150)		*p* = 0.0961	(*n* = 200)		<0.0001
*clk-1(qm30)*	20.7±3.9	32	36.8±7.4	58	+78%	23.0±3.9	30	+11%
	(*n* = 150)		(*n* = 200)		*p*<0.0001	(*n* = 100)		<0.0001
*daf-2(e1370)*	43.8±9.0	67	47.9±8.8	74	+9%	37.3±9.8	54	−15%
	(*n* = 150)		(*n* = 150)		*p* = 0.0002	(*n* = 100)		0.0006
*eat-2(ad1116)*	29.4±7.1	44	39.4±6.3	58	+34%	ND	ND	ND
	(*n* = 100)		(*n* = 150)		*p*<0.0001			
*daf-16(m26)*	16.7±2.3	21	22.6±3.6	28	+35%	ND	ND	ND
	(*n* = 100)		(*n* = 100)		*p*<0.0001			
*aak-2(ok524)*	17.3±2.5	23	22.4±4.2	30	+29%	ND	ND	ND
	(*n* = 100)		(*n* = 100)		*p*<0.0001			
*jnk-1(gk7)*	18.1±3.0	25	29.0±6.0	39	+60%	ND	ND	ND
	(*n* = 100)		(*n* = 100)		*p*<0.0001			
*wwp-1(ok1102)*	21.9±2.9	28	29.3±5.3*	37	+34%	ND	ND	ND
	(*n* = 100)		(*n* = 100)		*p*<0.0001			
*skn-1(zn67)*	17.6±2.3	22	22.8± 2.8*	25	+30%	ND	ND	ND
	(*n* = 50)		(*n* = 50)		*p*<0.0001			
*hsf-1(y441)*	14.9±3.1	22	22.5±5.7	37	+51%	ND	ND	ND
	(*n* = 100)		(*n* = 100)		*p*<0.0001			
*hif-1(ia4)*	23.4±4.1	31	28.7±6.8	42	+23%	ND	ND	ND
	(*n* = 50)		(*n* = 50)		*p*<0.0001			

*For these genotypes paraquat was only applied after the worms reached adulthood because of lethal effects during development. They should be compared to the wild type (N2) treated only during adulthood (not shown in the table), whose lifespan is 25.8±4.8, which is a 39% increase compared to non-treated worms.

NAC had only a moderate effect on the lifespan of the insulin-signaling *daf-2* mutants (Figure 2E), suggesting that only a small part of the increased longevity of these mutants requires elevated mitochondrial superoxide. However, NAC fully abolished the increased lifespan of *sod-2* mutants (Figure 2F), suggesting that, although increased generation of superoxide and other ROS as detected by our techniques were not significantly altered in these mutants, their increased lifespan depends on an elevation of superoxide or some other ROS. NAC did not shorten the lifespan of *clk-1* mutants at 10 mM (Figure 2D), or even at 15 mM (Table S1), indicating that ROS metabolism is relatively irrelevant to the aging phenotype of these mutants. The effect of NAC on the lifespan of *eat-2* could not be scored because NAC treatment rendered the animals unable to lay their eggs and they died from internal hatching at a young age. The origin of this effect is unknown. We also could not score the effect of NAC on RNAi-treated worms because 10 mM NAC was excessively damaging to the dsRNA-producing bacterial strain (HT115).

### Treatment With PQ at Low Concentration (0.1 mM) Increases Lifespan

**Figure 3:**
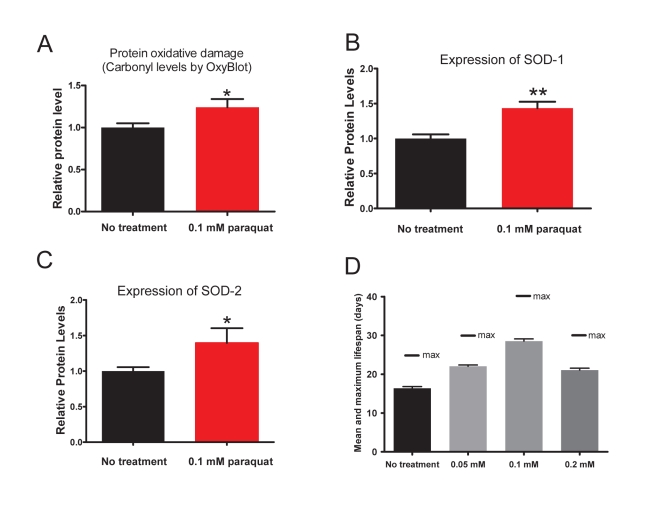
Treatment with 0.1 mM of paraquat (PQ) increases protein oxidative damage, superoxide dismutase expression, and lifespan. (A) Young wild type adults treated with 0.1 mM PQ have higher protein oxidative damage compared to untreated control. (B) Young wild type adults treated with 0.1 mM PQ since hatching express significantly more SOD-1 protein than untreated animals. (C) Young wild type adults treated with 0.1 mM paraquat since hatching express significantly more SOD-2 protein compared to untreated wild type worms. (D) Treatment with 0.05, 0.1, or 0.2 mM PQ increases mean and maximum lifespan significantly (see also Tables 1 and S1). *<0.05, ** *p*<0.001.

**Figure 4:**
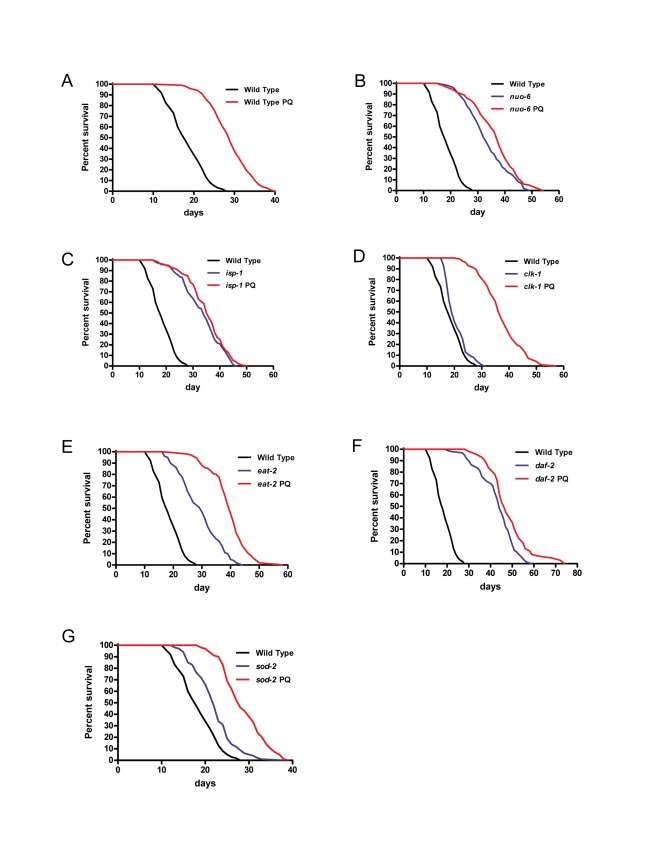
Lifespans of wild-type animals and mutants treated with 0.1 mM paraquat (PQ). The treatment had a dramatic lifespan-lengthening effect on the wild type (A) but no effect on the *nuo-6* (B) and *isp-1* (C) respiratory chain subunit mutants. In contrast, the treatment had a dramatic additive effect on the long-lived *clk-1* (D) and *eat-2* (E) mutants. The treatment has only a very moderate effect on *daf-2* mutants (F) but a strong effect on *sod-2* mutants (G). See Tables 1 and S1 for details of genotype, sample size, and statistical analysis.

To determine whether an elevation in mitochondrial superoxide generation is sufficient to increase lifespan, we used the superoxide generator PQ. Treatment of *C. elegans* with high concentration of PQ (>0.2 mM) is severely deleterious. We thus first tested the ability of PQ to increase ROS damage in the animals at a very low concentration (0.1 mM). We found that this treatment indeed measurably increased the level of oxidative damage to proteins at the young adult stage as assessed by determination of protein carbonylation (Figure 3A) and increased the expression of both the main cytoplasmic (SOD-1) and the main mitochondrial (SOD-2) superoxide dismutases (Figure 3B and 3C). We then tested whether PQ could increase the lifespan of the wild type at three different concentrations (0.05, 0.1, and 0.2 mM) and found that at all three concentrations both the mean and maximum lifespan were increased, with a maximal effect at 0.1 mM (Figures 3D and 4A, and Tables 1 and S1). The effect of 0.2 mM was less pronounced than that of 0.1 mM and similar to that of 0.05 mM, likely because at 0.2 mM a toxic effect starts to balance the pro-longevity effect. The effect does not depend on the exact chemical structure of paraquat, as benzyl-viologen, a compound with similar activity as PQ but structurally different, also increases lifespan (Table S1). A small effect of the prooxidant juglone under different conditions has also been documented previously [56]. The effect did not depend on an effect of PQ on the *E. coli* (OP50) food source, as the effect was also observed with heat-killed cells (Table S1). Finally, the effect was not confined to development or adulthood as PQ prolongs lifespan whether provided only during adult lifespan or only during development (Table S1).

PQ treatment failed to significantly prolong the lifespan on *nuo-6* and *isp-1* mutants (Figure 4B and 4C, and Tables 1 and S1). This experiment is equivalent to genetic epistasis analysis and suggests that *nuo-6*, *isp-1*, and PQ increase lifespan by the same mechanism. It also suggests that the maximum level of lifespan extension that can be obtained by increasing mitochondrial superoxide generation is already reached in these two mutants and further increase of superoxide generation through PQ treatment cannot increase lifespan further. This was not due to a resistance of these mutants to PQ as 0.2 mM PQ shortened the lifespan of the two mutants (Table S1). *sod-2* mutants, whose longevity is suppressed by NAC, are not as long-lived when untreated as wild type animals that are treated with PQ. However, treatment with PQ makes the *sod-2* mutants live as long as wild type animals treated with PQ (Figure 4G). This absence of additivity suggests that the longevity of *sod-2* mutants is indeed due to a small increase in superoxide, as expected from the function of SOD-2, and the suppressing effect of NAC on the mutant lifespan. In contrast to what we observed with *nuo-6*, *isp-1*, and *sod-2*, PQ treatment dramatically enhanced the lifespan of *clk-1* and *eat-2* mutants, significantly beyond the longevity increases induced by the mutations alone or by PQ applied to the wild type (Figure 4D and 4E, and Tables 1 and S1). This indicates that the effects of these mutations and the effect of superoxide are mechanistically distinct and additive, as expected from the finding that *clk-1* and *eat-2* mutants did not show increased mitochondrial superoxide levels (Figure 1F) and that the lifespan of *clk-1* mutants could not be shortened by NAC treatment (Figure 2D). PQ treatment had only a small lifespan-lengthening effect on *daf-2* (Figure 4F, and Tables 1 and S1), which is consistent with the finding that *daf-2* mutants already show a substantial increase in superoxide generation.

### NAC and PQ Treatments Do Not Affect Other Mitochondrial Parameters in a Manner That Could Predict Their Effect on Lifespan

**Figure 5:**
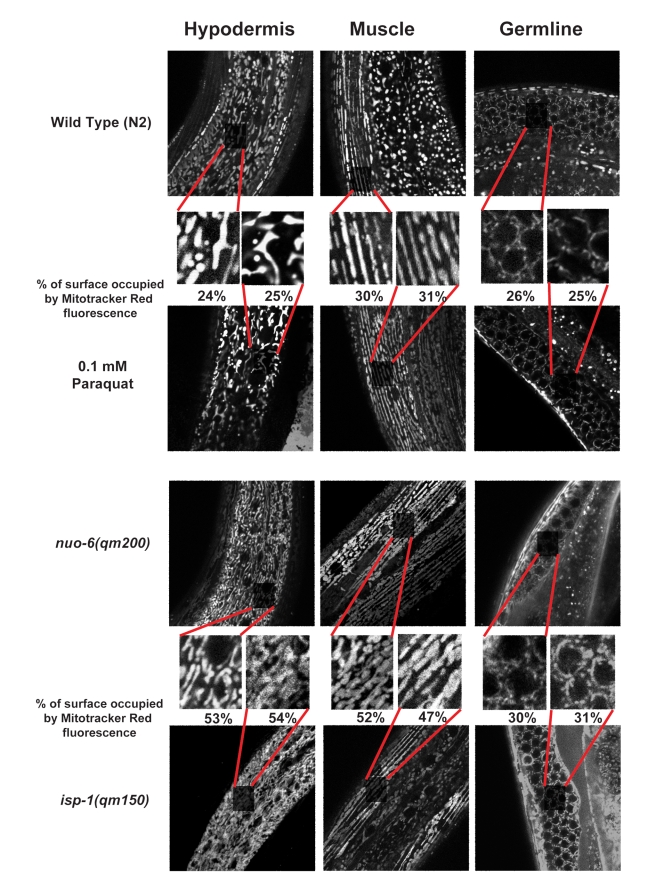
Treatment with 0.1 mM PQ does not affect mitochondrial abundance. Worms were treated with Mitotracker Red at 50 nM (final concentration) in M9 buffer for 20 min. All pictures were taken by confocal microscopy at 400×. A scale bar of 20 µm is shown in the upper right corner of the figure. For each genotype/treatment three tissues (hypodermis, muscle, and germline) were selected, and for each tissue at least five pictures from different worms were taken. An equal section of each picture was enlarged for quantitative comparisons. The percentage of surface occupied by mitochondria as stained by Mitotracker Red was measured and related to the total area of the selected region. A representative example for each tissue and condition is shown in the figure. The quantification for each sample is also shown in the figure below the enlarged areas. The sample size for the hypodermis was >15, and it was 5 for muscles and the germline. In muscles and in the hypodermis, the difference between the ETC mutants and the wild type was significant (*p*<0.001), while the difference between PQ treatment and untreated wild type worms was not. Thus, the *nuo-6* and *isp-1* mutations, but not treatment with 0.1 mM PQ, affect mitochondrial abundance.

We sought to determine whether the mutations and the PQ treatment had other common effects on mitochondrial function that could be responsible for the increased lifespans, besides elevation of superoxide levels. Work in other systems has suggested that increased mitochondrial biogenesis could impact lifespan positively [57],[58],[59], and mitochondrial defects in *C. elegans* have been found to stimulate mitochondrial biogenesis, resulting in a denser mitochondrial network [13]. We have examined mitochondrial density in the two mitochondrial mutants and in PQ-treated worms with Mitotracker Red, which is specific to mitochondria in mammalian cells [60],[61], which stains worms uniformly, and whose staining fully overlaps with that of mitochondrially targeted green fluorescent protein (GFP) (Figure S3). We found that *isp-1* and *nuo-6* display a denser mitochondrial network, as expected (Figure 5). However, this was not observed in wild type worms treated with PQ (Figure 5), indicating that the mechanism by which the superoxide triggers longevity does not require increased mitochondrial biogenesis. We also tested the effects of PQ and NAC treatment on oxygen consumption and ATP levels in the wild type and in the two mitochondrial mutants (Figure S4). NAC treatment increased oxygen consumption in the wild type and in the mutants. This result uncouples oxygen consumption from lifespan as NAC has no effect on the lifespan of the wild type, and its effect on the oxygen consumption of *isp-1* mutants is larger than on that of *nuo-6* mutants, although its effect on aging is smaller. PQ had an effect only on *nuo-6*, and it was small. Thus the effect of PQ on oxygen consumption also did not mirror its effect on lifespan. For ATP levels the only effect observed was a reduction by PQ of the elevated ATP levels that are observed in *nuo-6* mutants.

### PQ Is Able to Considerably Lengthen the Lifespan of a Variety of Mutants That Define Genetic Pathways of Aging

*daf-2* mutants have elevated superoxide levels, and they are sensitive to NAC (lifespan shortening by 15%). However, the level of superoxide in *daf-2* appears not to be sufficient for a maximal effect as these mutants remain somewhat sensitive to PQ (lifespan lengthening by 9%). To further study how superoxide plays a role in the lifespan of *daf-2* we studied genes that function downstream of *daf-2*. At least three genes are known to be required for the full lifespan extension of *daf-2*, that is, *daf-16*, *aak-2*, and *hsf-1* [62],[63],[64]. If one of these genes were necessary for an activity that mediates the small effect of PQ on *daf-2* mutants, PQ should not be able to prolong the lifespan of mutants of such a gene. In fact, however, we found that PQ prolonged the lifespan of all three mutants tested (Table 1). The lifespan increase upon PQ treatment of *daf-16* (35% increase) and *aak-2* (29% increase) is not as large as upon treatment of the wild type (58% increase). This suggests that part but not all of the lifespan increase determined by superoxide requires *daf-16* and *aak-2*. These findings are consistent with the observations that the lifespan extension provided by *nuo-6* and *daf-2(e1370)* are only partially additive (Table S1), similarly to what was found previously for *isp-1* and *daf-2* [12], and that elimination of *daf-16* partially shortens the lifespan of *isp-1* [12].

We also tested the sensitivity to PQ of mutants that are diagnostic of a variety of pathways of aging. In particular mutants of genes that, based on their known functions in *C. elegans* or that of their homologues in other systems, might encode the targets of superoxide signaling or be otherwise necessary for implementing superoxide signaling. The c-Jun N-terminal kinase 1 (JNK-1) is involved in stress responses in vertebrate cells and is a positive regulator of DAF-16 that acts in parallel to the effect of *daf-2* on *daf-16* [65]. We treated *jnk-1(gk7)* mutants with PQ and obtained a particularly large lifespan increase (Table 1). Although it is not clear what activities lie upstream of *jnk-1* in *C. elegans* nor whether it has other targets than *daf-16*, its activity does not appear necessary for the effect of superoxide. The transcription factor SKN-1 defends against oxidative stress by mobilizing the conserved phase II detoxification response and can delay aging independently of DAF-16 [66]. Although PQ induces oxidative stress and induces enzymes that protect from oxidative stress (Figure 3), it was still able to prolong the lifespan of *skn-1(zn67)* mutants (Table 1), indicating that *skn-1* does not act downstream of superoxide. *wwp-1* encodes a conserved E3 ubiquitin ligase that is necessary for lifespan extension by dietary restriction [67]. Treatment of *wwp-1(ok1102)* with PQ prolonged lifespan of these mutants, which is consistent with our finding that PQ can considerably extend the lifespan of *eat-2* mutants (Figure 4E). This confirmed that the lifespan increase produced by the superoxide increase in mitochondrial mutants is distinct from the mechanisms that support the lifespan effects of dietary restriction [14]. *hif-1* encodes a worm homologue of the vertebrate hypoxia inducible factor 1α (HIF-1α), a transcription factor involved in a number of protective mechanisms. In *C. elegans hif-1* is necessary for a lifespan pathway that involves proteolysis and that is distinct from insulin signaling [68] and has also been involved in the dietary restriction pathway [69]. In vertebrates HIF-1α is positively regulated by mitochondrial ROS [34],[35], which would make it an interesting candidate to mediate the effects of superoxide. However, PQ was fully capable of increasing the lifespan of the *hif-1* mutants (Table 1).

Several of the genes whose mutants remain sensitive to PQ, including *daf-16*, have been involved in stress responses, including oxidative stress, yet they do not seem necessary for the effect of PQ. Similarly we have shown previously that although the expression of SOD-1 and SOD-2 are elevated in *isp-1(qm150)* mutants, the elevation is not necessary for the extended lifespan of these mutants [24]. *nuo-6(qm200)* mutants also show elevated SOD-1 and SOD-2 expression [14], but this too is unnecessary for the longevity of the mutants, as RNAi against *sod-1* an *sod-2*, which we have shown to be efficient in reducing enzyme levels [24], does not shorten the lifespan of *nuo-6* mutants (Figure S5). We conclude that the mitochondrial mutants protect from an aspect of the aging process that has not yet been studied through mutants that affect stress. In addition, our observations suggest that the lifespan effect we observed is not hormetic, as neither superoxide-detoxifying enzymes, nor the regulatory factors that are involved in protection from oxidative stress, are crucially implicated.

## Discussion

We have shown previously that mutations in *isp-1* and *nuo-6* prolong lifespan by a common mechanism [14]. Using direct measurement of ROS and superoxide we find here that this mechanism involves an increase in mitochondrial superoxide generation that is necessary and sufficient for the longevity of these mutants. As ROS, including superoxide [70],[71],[72], are known to be intracellular messengers, the increased superoxide might trigger a signal transduction pathway that ultimately results in changes in nuclear gene expression [23]. Superoxide is highly reactive and could trigger such a signal by modifying proteins in the mitochondria or in the nearby cytosol after having escaped from the mitochondria through an appropriate channel [73],[74]. Although no superoxide sensor has yet been identified, a similar type of mechanism, in which a highly reactive, quickly diffusing, molecule modifies a signal transduction protein, has been evidenced for nitric oxide (NO), which covalently and permanently modifies guanylyl cyclases. Similarly, hydrogen peroxide (H_2_O_2_), the product of superoxide dismutation, can inactivate phosphatases involved in signal transduction. Future work will aim at using forward and reverse genetic screens in *C. elegans* to uncover the molecular machinery that reacts to the superoxide signal, as well as the transcription factors that are needed to regulate nuclear gene expression in response to the pathway's activation. In addition, the pattern of gene expression that results in increased lifespan in these mutants could be defined very specifically by identifying changes in the gene expression patterns that are common to *isp-1*, *nuo-6,* and PQ treatment and that are suppressed by treatment with NAC.

A number of studies in *C. elegans* have explored hormesis by treating animals with sub-lethal but clearly deleterious treatments for a short period of time and observing subsequent prolongation of lifespan [75]. These hormetic effects are different from what we have observed and describe here, as both the genetic mutations and the very low level PQ are present throughout life and as only a part of the effect we observe might require the insulin signaling pathway. Furthermore, although in *nuo-6* and *isp-1* mutants the expression levels of the superoxide dismutases SOD-1 and SOD-2 are elevated, likely in response to the elevated superoxide generation, and as one expects in the hormetic response, these elevations are not necessary for the lifespan prolongation of *nuo-6* (Figure S5) or *isp-1* [24].

CLK-1 is a mitochondrial protein that is required for ubiquinone biosynthesis and its absence affects mitochondrial function [50], although it could potentially affect many other processes as ubiquinone is found in all membranes. Furthermore, ubiquinone is both a prooxidant as co-factor in the respiratory chain and an anti-oxidant. Interestingly, the mechanism of lifespan prolongation induced by *clk-1* appears to be entirely distinct from, but particularly synergistic with, that induced by elevated superoxide. Indeed, *clk-1* mutants do not show elevated superoxide generation and are not affected by NAC. Furthermore, although double mutant combinations of *clk-1* with *nuo-6* and *isp-1* are not viable (unpublished data) the lifespans of *clk-1* mutants treated with PQ (Figure 4D), or of *sod-2;clk-1* mutants [26], or of *clk-1;daf-2* mutants [76] are much greater than expected from simple additivity of the effects of individual mutations or treatments.

Studies in yeast [77] and in worms [78] have suggested that an increase in ROS from mitochondria might also be important in triggering the lifespan extension produced by glucose restriction. However, our results here with an *eat-2* mutation, one of the ways in which global dietary restriction can be produced in worms, as well as with a *wwp-1* and *hif-1*, which may function downstream of dietary restriction, did not reveal an involvement of superoxide signaling, providing further evidence for a distinction between the mechanisms of glucose restriction and dietary restriction. It remains possible, however, that DR could lead to superoxide or ROS production when it is induced by other methods than the use of an *eat-2* mutant, as it is well documented that different types of DR induce different molecular mechanisms [79].

One question that our current experiments do not address is whether the mitochondrial dysfunction in the mutants, or the effect of PQ, is necessary in every tissue in order to increase longevity. There are indications for both the insulin signaling pathway mutants [80],[81] and dietary restriction [67],[82] that the entire effect might be mediated by action in particular cells that influence the physiology of the whole organism. Similarly, the presence or absence of the germline is sufficient for a dramatic effect on lifespan [83]. For mitochondrial dysfunction the question could be addressed in the future by mosaic analysis and by purifying and analyzing mitochondria from specific tissues using our flow cytometry technique to purify mitochondria expressing GFP in a tissue-specific manner.

The oxidative stress theory of aging has been one of the most acknowledged theories of aging for the simple reason of the strikingly good correlation between the levels of oxidative stress and the aged phenotype [8]. A number of recent results in worms and in mice, however, have suggested that oxidative stress cannot be the cause of aging [24],[25],[26],[30]. Our findings suggest a conceptual framework for why oxidative stress and the aged phenotype are so tightly correlated [31]. In this model mitochondria, like the rest of the cell, sustain a variety of age-dependent insults (not only and not even principally from oxidative stress) that trigger an increase in superoxide, which acts as a signal that induces general protective and repair mechanisms. However, aging in most animals is clearly irreversible, indicating that the protective mechanisms, which must have evolved to control damage in young organisms, are unable to fully prevent the accumulation of age-related damage. Thus, as superoxide is a reactive molecule as well as a signal, and as age-dependent damage cannot be fully reversed, it is possible that at high ages the chronically elevated superoxide will participate in creating some of the damage itself. This could explain the strong tendency for aged animals to have high oxidative stress and high oxidative damage, although it does not imply that ROS cause aging or even that they are a major source of age-dependent damage. In this model, the *nuo-6* and *isp-1* mutations lead to increased longevity because they turn on the stress signal prematurely and thus slow down the entire process.

## Materials and Methods

### Lifespan Scoring

Eggs were placed on plates at 20°C and left for 1 h to hatch. Larvae that had hatched during that period were placed onto fresh plates and monitored once daily until death. The animals were transferred once daily while producing eggs to keep them separate from their progeny. Animals were scored as dead when they no longer responded with movement to light prodding on the head and tail. Missing worms and worms that have died because of internal hatching (bagging) were replaced from a backup group. Survival was scored every day.

### Drug Treatment

Drugs were added into NGM media from a high concentration stock solution (500 mM for NAC, 1 M for PQ, and 500 mM for vitamin C) before pouring of the plates. Plates were made fresh each week. Gravid adult worms were transferred from normal NGM plates to drug plates and left to lay eggs for 3 h. With each transfer of worms a substantial amount of bacteria was also transferred onto the new plates. The progeny was then scored for different phenotypes.

### Staining and Confocal Imaging

All dyes except MitoSox were diluted in DMSO at high concentration (all at 5 mM except H_2_DCFDA, which is at 10 mM) and frozen at −20°C as a stock. MitoSox was prepared fresh at 5 mM for each use. Before staining stocks were diluted in M9 buffer at a 1∶1000 dilution. Young adult worms were transferred into staining solution and stained for 20 min. Worms were mounted on a thick layer of half-dried agar pad on microscopic glass slides and then subjected to confocal microscopy (Zeiss LSM 510 Meta). Pictures were taken by Zeiss LSM Imaging software and analyzed by Volocity V4.0 software.

### Oxygen Consumption

Five young adult worms (1st day of adulthood) were placed into 0.25 µl M9 buffer in a 0.5 µl sealed chamber at 22°C. A fiber optical oxygen sensor (AL300 FOXY probe from Ocean Optics) was inserted into this chamber and oxygen partial pressure was monitored for 15 to 30 min. Oxygen consumption measured in this way was normalized to body volume. For this worms were photographed at each measurement day under a binocular microscope and their cross-section was calculated with ImageJ software. Worm volume was determined by the formula: volume (nl)  = 1.849 • 10–7 (nl/µm3) • area 1.5 (µm3) [84].

### Expression Levels of Superoxide Dismutases (SODs)

After RNAi treatment, 100 young adult worms of each genotype were picked, lysed in 2× loading buffer, and subjected to electrophoresis in 12% SDS–polyacrylamide gels (SDS–PAGE), and then blotted onto nitrocellulose membrane (Bio-Rad). After applying primary antibody (1∶1000, rabbit polyclonal antibody against worm SOD-1 or SOD-2) and secondary antibody (1∶10,000 mouse anti-rabbit IgG, Invitrogen), the membranes were incubated with the ECL plus detection reagent (Amersham Biosciences) and scanned using a Typhoon trio plus scanner. Band densities were analyzed by ImageQuant TL V2003.03.

### Fluorescence Activated Cell Sorting

For fluorescence activated cell sorting [37], adult worms grown on large NGM plates were collected and washed 3 times with M9 buffer. Worms were then suspended in 5× isolation buffer (200 mM mannitol; 120 mM sucrose; 10 mM Tris; 1 mM EGTA; pH 7.4) and set on ice. Worms were broken up with a 5 ml glass-glass homogenizer and centrifuged at 600 g for 10 min, the supernatant was collected and re-centrifuged at 7,800 g for 10 min, and the pellet was washed once with isolation buffer and then suspended in isolation buffer and kept on ice. Different dyes were added from stocks into the analysis buffer (250 mM sucrose; 20 mM MOPS; 100 uM KPi; 0.5 mM MgCl2; 1 uM CsA pH 7.0) at a 1∶1000 dilution before staining. 100 µl of mitochondria was added to 900 µl of analysis buffer with dye and substrate and incubated for 1 h at room temperature. Mitochondria were recollected by 7,800 g centrifugation and then suspended in 500 µl analysis buffer. A FACSCalibur instrument equipped with a 488 nm Argon laser and a 635 nm red diode laser (Becton Dickinson) was used. Data from the experiments were analyzed using the CellQuest software (Becton Dickinson). To exclude debris, samples were gated based on light-scattering properties in the SSC (side scatter) and FSC (forward scatter) modes, and 20,000 events per sample were collected, using the “low” setting for sample flow rate (Figure S1).

### ATP Content

200 age-synchronized young adult worms were collected in M9 buffer and washed three times. Worm pellets were treated with three freeze/thaw cycles and boiled for 15 min to release ATP and destroy ATPase activity, and then spun at 4°C and 11,000 g for 10 min. ATP contents were measured with a kit (Invitrogen, Carlsbad, California, USA; Cat: A22066). The ATP content value was then normalized to the soluble protein level of the same preparation, measured with the protein assay from Bio-Rad.

### Dyes Used for Staining and FACS

Mitotracker green (Invitrogen M7514) stock concentration 5 mM; H2DCFDA (Invitrogen D399) stock concentration 10 mM; Mitotracker red (Invitrogen M7512) stock concentration 5 mM.

## Supporting Information

Figure S1 **Selection of isolated mitochondria and ROS-sensitive dye analysis.** Mitochondria were prepared as described in Materials and Methods. (A) A FACSCalibur flow cytometry cell sorter from Becton Dickinson equipped with a 488 nm Argon laser and a 635 nm red diode laser was used. Data from the experiments were analyzed using the CellQuest software (Becton Dickinson). To exclude debris, samples were gated based on light-scattering properties in the SSC (side scatter) and FSC (forward scatter) modes, and 20,000 events per sample within the region (gate) delimitated by a square in (A) were collected, using the “low” setting for sample flow rate. 99% of the particles in that region successfully stained with the mitochondria-specific dye Mitotracker Green. (B) Isolated mitochondria were incubated with analysis buffer containing substrate (see Materials and Methods) and MitoSox (1 µM) at room temperature for 1 h and then sorted and the fluorescence of mitochondria in the gate measured. The purple area represents un-stained control. Paraquat (red line) was able to increase ROS generation over the untreated control (green line), while NAC (blue line) decreased the superoxide signal. Note that the *x*-axis shows a log scale. (C) Isolated mitochondria (see A) from wild-type worms were stained with both H2DCF-DA (the signal plotted on FL1-H; 530±15 nm channel) and Mitotracker Red (the signal plotted on FL3-H; ≥670 nm channel). When particles were stained by both dyes (upper-right region), the signals were strongly correlated. Furthermore, 89.6%±2.4% (*n* = 4) of the particles stained by H2DCF-DA were also stained by Mitotracker Red.
(0.11 MB PDF)

Figure S2 **Absence of deleterious effects of N-acetyl-cysteine.** (A) NAC (N-acetyl-cysteine) had no effect on the apparent health of *isp-1* or *nuo-6* mutants. Mutant animals were treated or not with 10 mM NAC throughout their lives and all pictures in the panel were taken on day 23 of their lifespan, when less than 25% of untreated mutants but more than 75% of NAC-treated mutants had already died. NAC-treated *isp-1* and *nuo-6* mutants did not show any visible ill effects from the treatment. All worms are shown at the same magnification; scale bar is 0.5 mm. Phenotypes possibly resulting from NAC treatment of *nuo-6(qm200)* mutants were also quantified. We chose to examine *nuo-6* mutants because their longevity was the most sensitive to NAC (completely suppressed at 10 mM). Adult worms were allowed to lay eggs on NAC plates and phenotypes of the resulting F1 progeny were scored. (B) NAC significantly decreased defecation cycle length of the wild type (*p* = 0.0104), while it has no significant effect on that of *nuo-6(qm200)* mutant (*n* = 15). (C) NAC significantly increased post-embryonic development length of both the wild type and *nuo-6(qm200)* mutants (*n* = 100). (D) NAC has no significant effect on brood size of both the wild type and *nuo-6(qm200)* mutants (*n* = 50). (E) NAC has no significant effect on the swimming rate (frequency of thrashing) of the wild type, but it significantly increased that of *nuo-6(qm200)* mutants (*p* = 0.0024) (*n* = 15).
(0.06 MB PDF)

Figure S3 **Co-localization of Mitotracker Red and GFP signals in** ***C. elegans*** **mitochondria.** We used Mitotracker Red to stain worms carrying the transgene *qmIs16[Pclk-1::clk-1::gfp*], which expresses the mitochondrial protein CLK-1 fused to GFP [50]. Staining was as described in Materials and Methods. Worms were mounted on agar pads on slides and subjected to confocal microscopical analysis. (A) Mitotracker Red expression in hypodermal tissue. (B) The same region as in (A) expressing the mitochondrial GFP fusion. (C) The merged images of (A) and (B). The Mitotracker Red and GFP signals are co-localized.
(0.10 MB PDF)

Figure S4 **Effect of paraquat (PQ) and N-acetylcysteine (NAC) on energy metabolism.** Untreated wild type controls and animals treated with 0.1 mM PQ or 10 mM NAC since hatching were collected at the first day of adulthood for both experiments. (A) Animals in groups of 5 (n≥3) were transferred in 0.25 µl M9 buffer into a 0.5 µl chamber where oxygen concentration was measured with a fiber optic oxygen sensor (AL300 FOXY probe from Ocean Optics) for 15–30 min. The body volume of animals was calculated from pictures of the same worms and used for normalization. PQ had a small but significant consumption-increasing effect only on *nuo-6* mutants. NAC increased oxygen consumption in all three genotypes, with the largest effect on *isp-1* mutants. (B) The ATP content from 200 worms was normalized to the amount of soluble protein from the same sample (*n*≥6). Both PQ and NAC treatment had no effect on ATP content with the exception of PQ-treated nuo-6mutants, in which the treatment suppressed the high ATP content that is observed in the untreated animals. For all statistic analyses we used the Student's *t* test.* *p*<0.05, ** *p*<0.01 and *** *p*<0.001.
(0.06 MB PDF)

Figure S5 **SOD-1 and SOD-2 are not necessary for the longevity of** ***nuo-6(qm200)*** **.** Knocking down *sod-1* (red) or *sod-2* (blue) does not shorten the long lifespan of *nuo-6(qm200)* mutant. In fact silencing these two genes slightly increases the lifespan of *nuo-6* mutants. Mean lifespan of control (empty vector) is 33 d (green), mean lifespan after *sod-1* RNAi treatment is 35 d, and mean lifespan after *sod-2* RNAi treatment is 36.5 d; *p*<0.05 for both RNAi experiments compared to control, analyzed by curve comparison using the log-rank test.
(0.02 MB PDF)

Table S1 **Individual aging experiments and statistics**.
(0.14 MB PDF)

## Abbreviations

GFP: green fluorescent protein
HIF: hypoxia-inducible factor
JNK-1: c-Jun N-terminal kinase 1
NAC: N-acetyl-cysteine
PQ: paraquat
ROS: reactive oxygen species
SOD: superoxide dismutase
