# Supplementary material for: A Mitochondrial Superoxide Signal Triggers Increased Longevity in Caenorhabditis elegans
Source: PLoS Biol. 2010 Dec 7;8(12):e1000556. doi: 10.1371/journal.pbio.1000556 (PMC2998438; doi:10.1371/journal.pbio.1000556)
Supplement: Figure S1 — Selection of isolated mitochondria and ROS-sensitive dye analysis. Mitochondria were prepared as described in Materials and Methods. (A) A FACSCalibur flow cytometry cell sorter from Becton Dickinson equipped with a 488 nm Argon laser and a 635 nm red diode laser was used. Data from the experiments were analyzed using the CellQuest software (Becton Dickinson). To exclude debris, samples were gated based on light-scattering properties in the SSC (side scatter) and FSC (forward scatter) modes, and 20,000 events per sample within the region (gate) delimitated by a square in (A) were collected, using the “low” setting for sample flow rate. 99% of the particles in that region successfully stained with the mitochondria-specific dye Mitotracker Green. (B) Isolated mitochondria were incubated with analysis buffer containing substrate (see Materials and Methods) and MitoSox (1 µM) at room temperature for 1 h and then sorted and the fluorescence of mitochondria in the gate measured. The purple area represents un-stained control. Paraquat (red line) was able to increase ROS generation over the untreated control (green line), while NAC (blue line) decreased the superoxide signal. Note that the x-axis shows a log scale. (C) Isolated mitochondria (see A) from wild-type worms were stained with both H2DCF-DA (the signal plotted on FL1-H; 530±15 nm channel) and Mitotracker Red (the signal plotted on FL3-H; ≥670 nm channel). When particles were stained by both dyes (upper-right region), the signals were strongly correlated. Furthermore, 89.6%±2.4% (n = 4) of the particles stained by H2DCF-DA were also stained by Mitotracker Red. (0.11 MB PDF) [file pbio.1000556.s001.pdf]

Supporting Figure 1_Yang and Hekimi

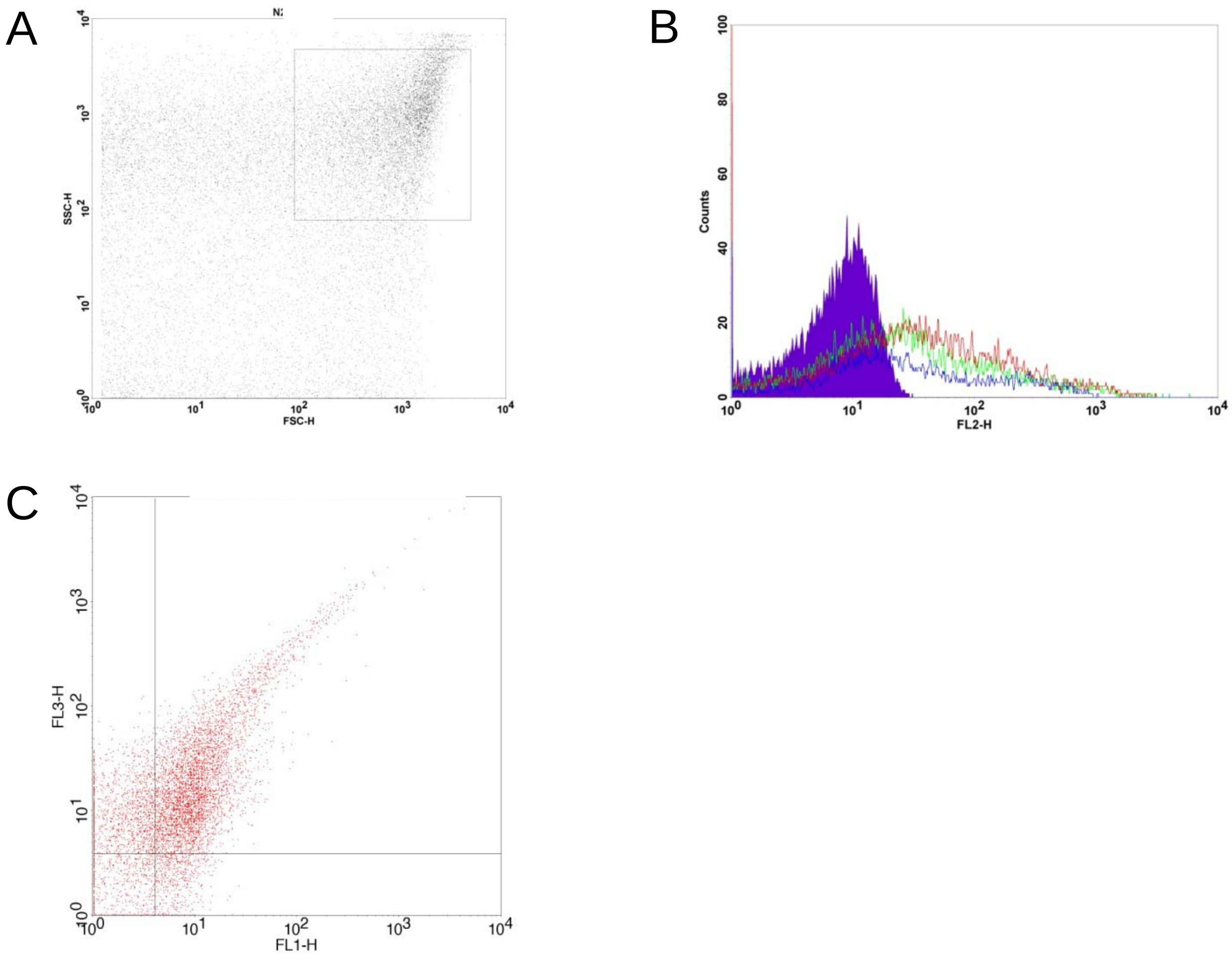

Selection of isolated mitochondria and ROS-sensitive dye analysis. Mitochondria were prepared as described in Material and Methods. A: A FACSCalibur flow cytometry cell sorter from Becton Dickinson equipped with a 488-nm Argon laser and a 635-nm red diode laser was used. Data from the experiments were analyzed using the CellQuest software (Becton Dickinson). To exclude debris, samples were gated based on light-scattering properties in the SSC (side scatter) and FSC (forward scatter) modes, and 20,000 events per sample within the region (gate) deliminated by a square in A. were collected, using the ”low” setting for sample flow rate. 99% of the particles in that region successfully stained with the mitochondria-specific dye Mitotracker Green. B. Isolated mitochondria were incubated with analysis buffer contain substrate (see Materials and Methods) and MitoSox (1μM) at room temperature for 1 hour and then sorted and the fluorescence of mitochondria in the gate measured. The purple area represents un-stained control. Paraquat (red line) was able to increase ROS generation over the untreated control (green line), while NAC (blue line) decreased the superoxide signal. Note that the X-axis shows a log scale. C. Isolated mitochondria (see A.) from wild-type worms were stained with both H_2DCF-DA (the signal plotted on FL1-H; 530 ± 15 nm channel) and Mitotracker Red (the signal plotted on FL3-H; ≥670nm channel). When particles were stained by both dyes (upper-right region), the signals were strongly correlated. Furthermore, 89.6 ± 2.4% (n=4) of the particles stained by H_2DCF-DA were also stained by Mitotracker Red.
