# Supplementary material for: A Mitochondrial Superoxide Signal Triggers Increased Longevity in Caenorhabditis elegans
Source: PLoS Biol. 2010 Dec 7;8(12):e1000556. doi: 10.1371/journal.pbio.1000556 (PMC2998438; doi:10.1371/journal.pbio.1000556)
Supplement: Figure S2 — Absence of deleterious effects of N-acetyl-cysteine. (A) NAC (N-acetyl-cysteine) had no effect on the apparent health of isp-1 or nuo-6 mutants. Mutant animals were treated or not with 10 mM NAC throughout their lives and all pictures in the panel were taken on day 23 of their lifespan, when less than 25% of untreated mutants but more than 75% of NAC-treated mutants had already died. NAC-treated isp-1 and nuo-6 mutants did not show any visible ill effects from the treatment. All worms are shown at the same magnification; scale bar is 0.5 mm. Phenotypes possibly resulting from NAC treatment of nuo-6(qm200) mutants were also quantified. We chose to examine nuo-6 mutants because their longevity was the most sensitive to NAC (completely suppressed at 10 mM). Adult worms were allowed to lay eggs on NAC plates and phenotypes of the resulting F1 progeny were scored. (B) NAC significantly decreased defecation cycle length of the wild type (p = 0.0104), while it has no significant effect on that of nuo-6(qm200) mutant (n = 15). (C) NAC significantly increased post-embryonic development length of both the wild type and nuo-6(qm200) mutants (n = 100). (D) NAC has no significant effect on brood size of both the wild type and nuo-6(qm200) mutants (n = 50). (E) NAC has no significant effect on the swimming rate (frequency of thrashing) of the wild type, but it significantly increased that of nuo-6(qm200) mutants (p = 0.0024) (n = 15). (0.06 MB PDF) [file pbio.1000556.s002.pdf]

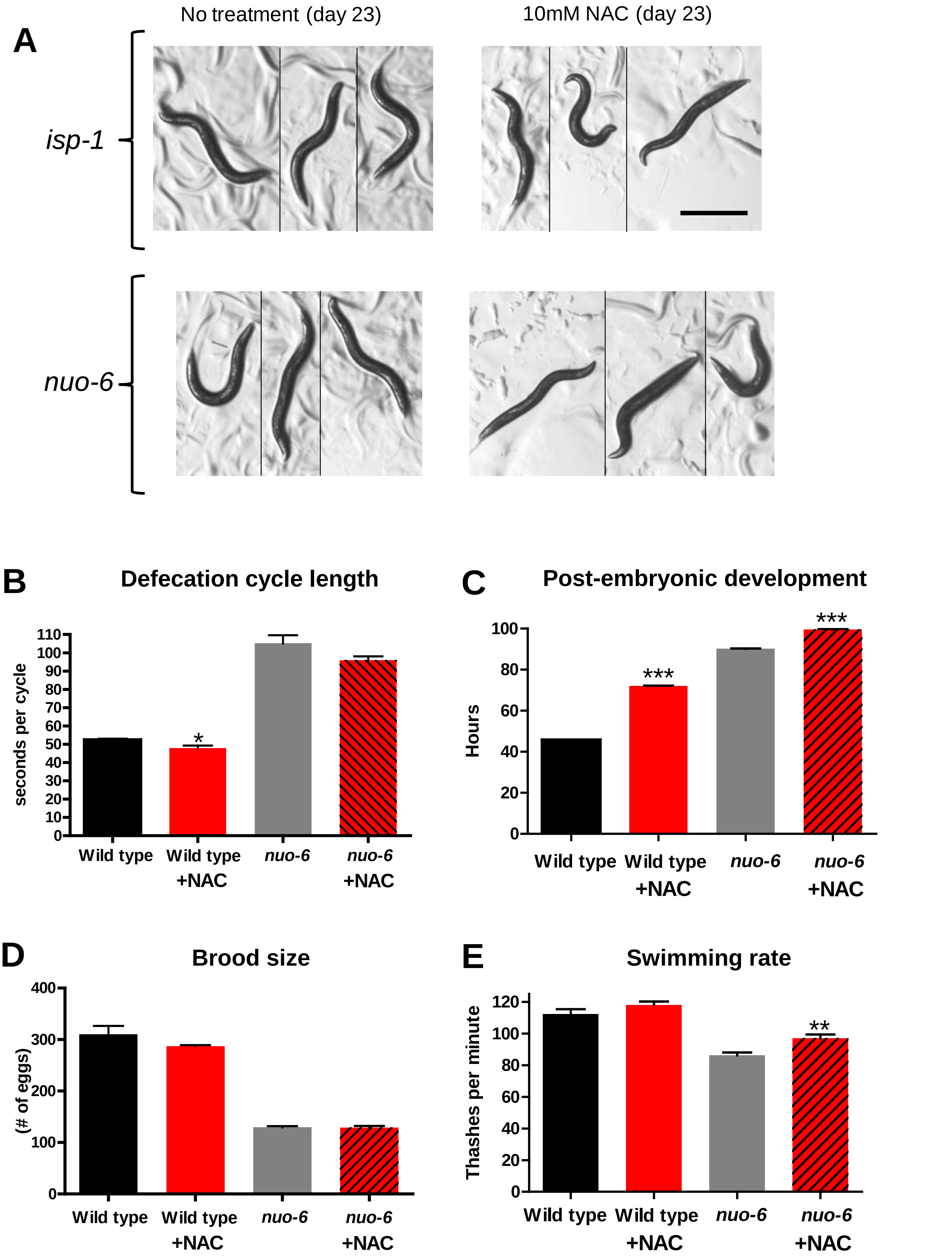

No treatment (day 23)
10mM NAC (day 23)
A
isp-1
nuo-6
B
Defecation cycle length
seconds per cycle
110
100
90
80
70
60
50
40
30
20
10
0
*
Wild type
Wild type +NAC
nuo-6
nuo-6 +NAC
C
Post-embryonic development
Hours
100
80
60
40
20
0
***
***
Wild type
Wild type +NAC
nuo-6
nuo-6 +NAC
D
Brood size
(# of eggs)
400
300
200
100
0
Wild type
Wild type +NAC
nuo-6
nuo-6 +NAC
E
Swimming rate
Thashes per minute
120
100
80
60
40
20
0
**
Wild type
Wild type +NAC
nuo-6
nuo-6 +NAC

Legend of Supporting Figure S2: **A**) NAC (N-acetyl-cysteine) had no effect on the apparent health of *isp-1* or *nuo-6* mutants. Mutant animals were treated or not with 10mM NAC throughout their lives and all pictures in the panel were taken on day 23 of their lifespan, when less than 25% of untreated mutants, but more than 75% of NAC-treated mutants had already died. NAC treated *isp-1* and *nuo-6* mutants did not show any visible ill effects from the treatment. All worms are shown at the same magnification; scale bar is 0.5mm. Phenotypes possibly resulting from NAC treatment of *nuo-6(qm200*) mutants were also quantified. We chose to examine *nuo-6* mutants because their longevity was the most senstivie to NAC (completely suppressed at 10mM). Adult worms were allowed to lay eggs on NAC plates and phenotypes of the resulting F1 progeny were scored. **B**) NAC significantly decreased defecation cycle length of the wildtype (p=0.0104), while it has no significant effect on that of *nuo-6(qm200)* mutant (n=15). **C**) NAC significant increased post-embryonic development length of both the wild type and *nuo-6(qm200)* mutants (n=100). **D**) NAC has no significant effect on brood size of both the wild type and *nuo-6(qm200)* mutants (n=50). **E**) NAC has no significant effect on the swimming rate (frequency of thrashing) of the wild type, but it significantly increased that of *nuo-6(qm200)* mutants (p=0.0024) (n=15).
