# Supplementary figures and images for: A Mitochondrial Superoxide Signal Triggers Increased Longevity in Caenorhabditis elegans
Source: PLoS Biol. 2010 Dec 7;8(12):e1000556. doi: 10.1371/journal.pbio.1000556 (PMC2998438; doi:10.1371/journal.pbio.1000556)

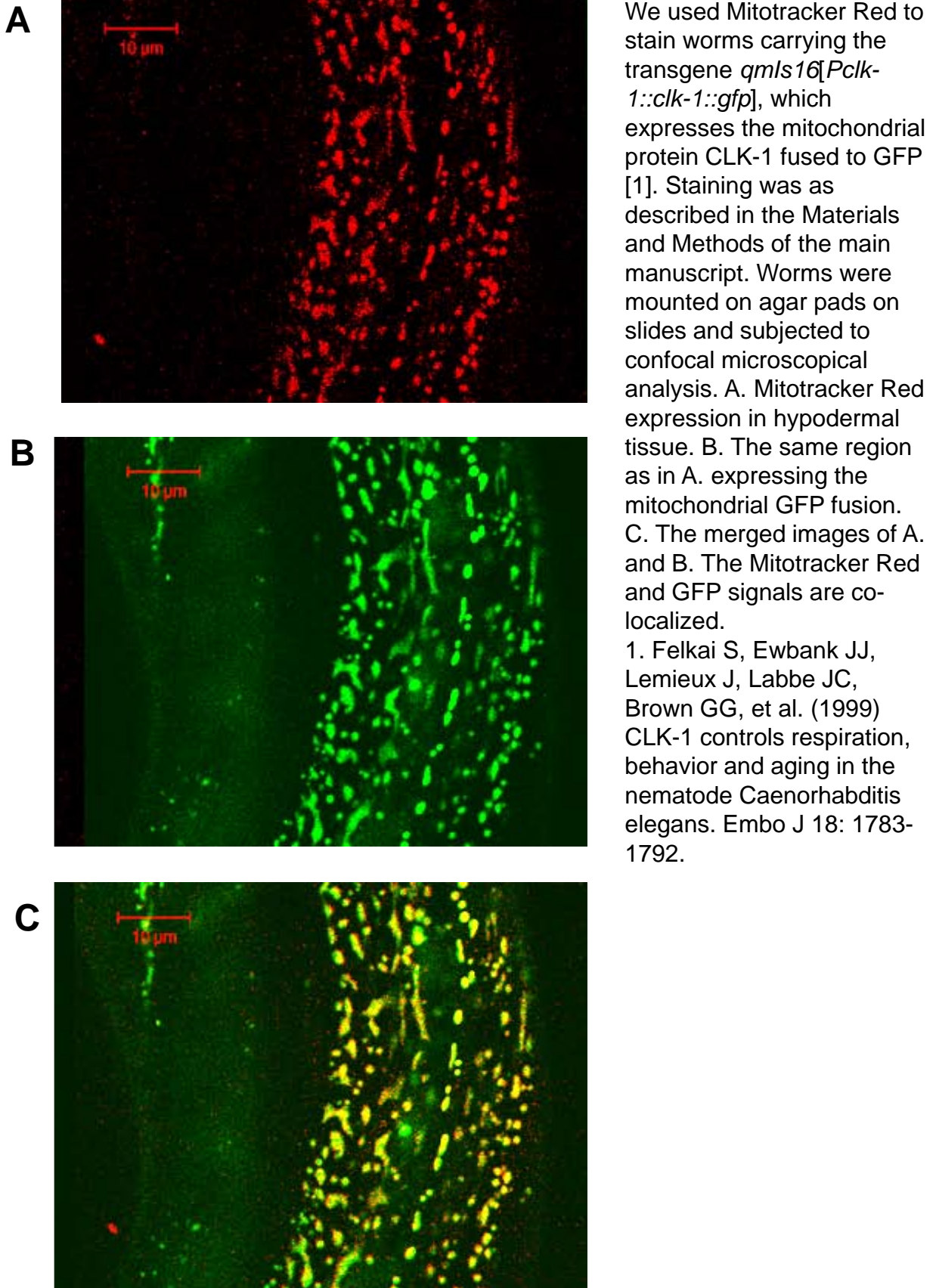

Supplement: Figure S3 — Co-localization of Mitotracker Red and GFP signals in C. elegans mitochondria. We used Mitotracker Red to stain worms carrying the transgene qmIs16[Pclk-1::clk-1::gfp], which expresses the mitochondrial protein CLK-1 fused to GFP [50]. Staining was as described in Materials and Methods. Worms were mounted on agar pads on slides and subjected to confocal microscopical analysis. (A) Mitotracker Red expression in hypodermal tissue. (B) The same region as in (A) expressing the mitochondrial GFP fusion. (C) The merged images of (A) and (B). The Mitotracker Red and GFP signals are co-localized. (0.10 MB PDF) [file pbio.1000556.s003.pdf]
