# Supplementary material for: A Mitochondrial Superoxide Signal Triggers Increased Longevity in Caenorhabditis elegans
Source: PLoS Biol. 2010 Dec 7;8(12):e1000556. doi: 10.1371/journal.pbio.1000556 (PMC2998438; doi:10.1371/journal.pbio.1000556)
Supplement: Figure S4 — Effect of paraquat (PQ) and N-acetylcysteine (NAC) on energy metabolism. Untreated wild type controls and animals treated with 0.1 mM PQ or 10 mM NAC since hatching were collected at the first day of adulthood for both experiments. (A) Animals in groups of 5 (n≥3) were transferred in 0.25 µl M9 buffer into a 0.5 µl chamber where oxygen concentration was measured with a fiber optic oxygen sensor (AL300 FOXY probe from Ocean Optics) for 15–30 min. The body volume of animals was calculated from pictures of the same worms and used for normalization. PQ had a small but significant consumption-increasing effect only on nuo-6 mutants. NAC increased oxygen consumption in all three genotypes, with the largest effect on isp-1 mutants. (B) The ATP content from 200 worms was normalized to the amount of soluble protein from the same sample (n≥6). Both PQ and NAC treatment had no effect on ATP content with the exception of PQ-treated nuo-6mutants, in which the treatment suppressed the high ATP content that is observed in the untreated animals. For all statistic analyses we used the Student's t test.* p<0.05, ** p<0.01 and *** p<0.001. (0.06 MB PDF) [file pbio.1000556.s004.pdf]

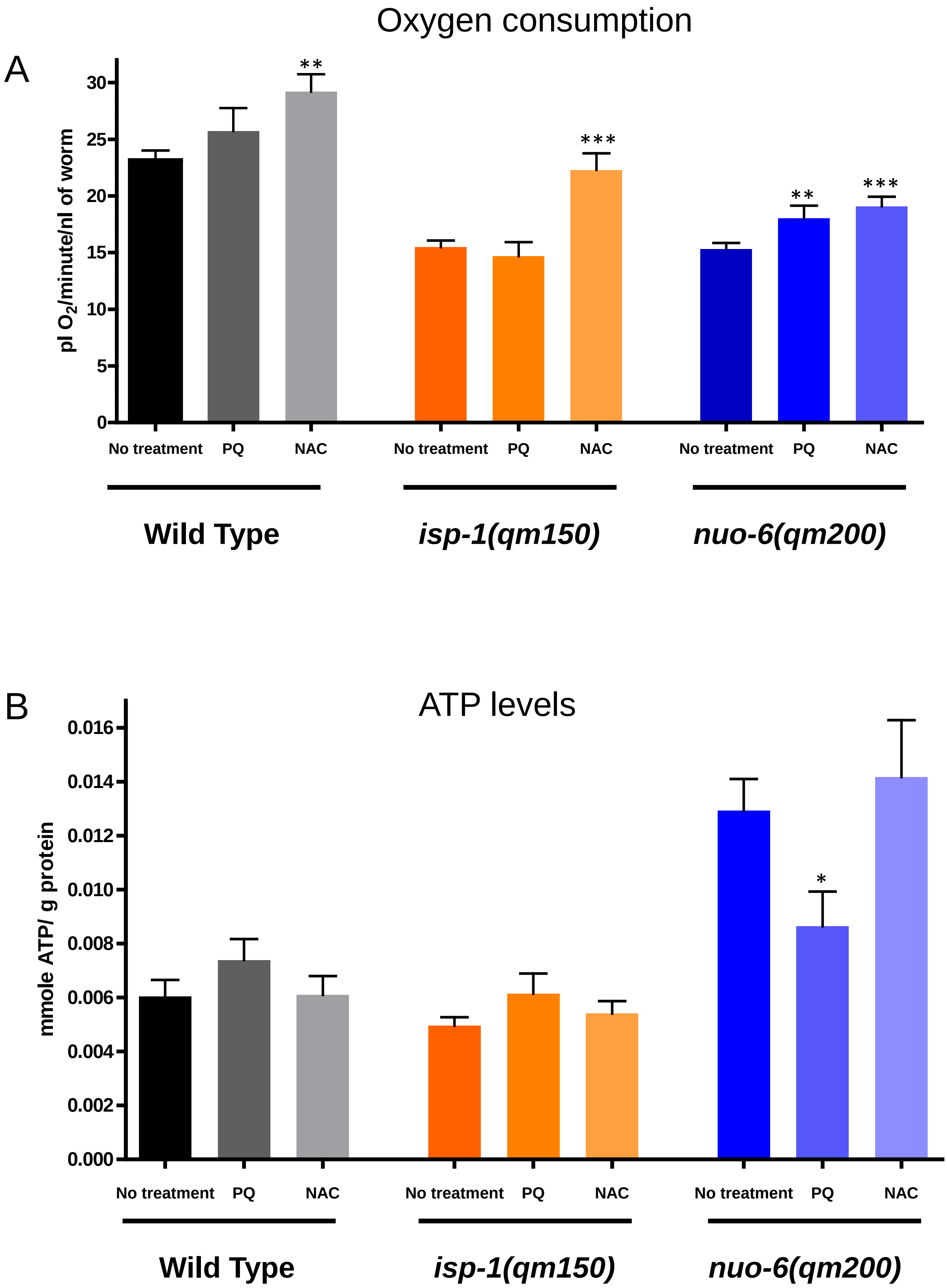
Oxygen consumption
A
pl O_2/minute/nl of worm
0
5
10
15
20
25
30
**
***
**
***
No treatment
PQ
NAC
No treatment
PQ
NAC
No treatment
PQ
NAC
Wild Type
isp-1(qm150)
nuo-6(qm200)
B
ATP levels
mmole ATP/ g protein
0.000
0.002
0.004
0.006
0.008
0.010
0.012
0.014
0.016
*
No treatment
PQ
NAC
No treatment
PQ
NAC
No treatment
PQ
NAC
Wild Type
isp-1(qm150)
nuo-6(qm200)

Effect of paraquat (PQ) and N-acetylcysteine (NAC) on energy metabolism. Untreated wild type controls and animals treated with 0.1mM PQ or 10mM NAC since hatching were collected at the first day of adulthood for both experiments. A: Animals in groups of 5 ($n \geq 3$) were transferred in 0.25μl M9 buffer into a 0.5μl chamber where oxygen concentration was measured with a fiber optic oxygen sensor (AL300 FOXY probe from Ocean Optics) for 15-30 minutes. The body volume of animals was calculated from pictures of the same worms and used for normalization. PQ had a small but significant consumption -increasing effect only on *nuo-6* mutants. NAC increased oxygen consumption in all three genotypes with the largest effect on *isp-1* mutants. B: The ATP content from 200 worms was normalized to the amount of soluble protein from the same sample ($n \geq 6$). Both PQ and NAC treatment had no effect on ATP content with the exception of PQ-treated *nuo-6* mutants, in which the treatment suppressed the high ATP content that is observed in the untreated animals. For all statistic analyses we used the Student t-test. * for $p<0.05$, ** for $p<0.01$ and *** for $p<0.001$
