# Supplementary material for: A Mitochondrial Superoxide Signal Triggers Increased Longevity in Caenorhabditis elegans
Source: PLoS Biol. 2010 Dec 7;8(12):e1000556. doi: 10.1371/journal.pbio.1000556 (PMC2998438; doi:10.1371/journal.pbio.1000556)
Supplement: Figure S5 — SOD-1 and SOD-2 are not necessary for the longevity of nuo-6(qm200) . Knocking down sod-1 (red) or sod-2 (blue) does not shorten the long lifespan of nuo-6(qm200) mutant. In fact silencing these two genes slightly increases the lifespan of nuo-6 mutants. Mean lifespan of control (empty vector) is 33 d (green), mean lifespan after sod-1 RNAi treatment is 35 d, and mean lifespan after sod-2 RNAi treatment is 36.5 d; p<0.05 for both RNAi experiments compared to control, analyzed by curve comparison using the log-rank test. (0.02 MB PDF) [file pbio.1000556.s005.pdf]

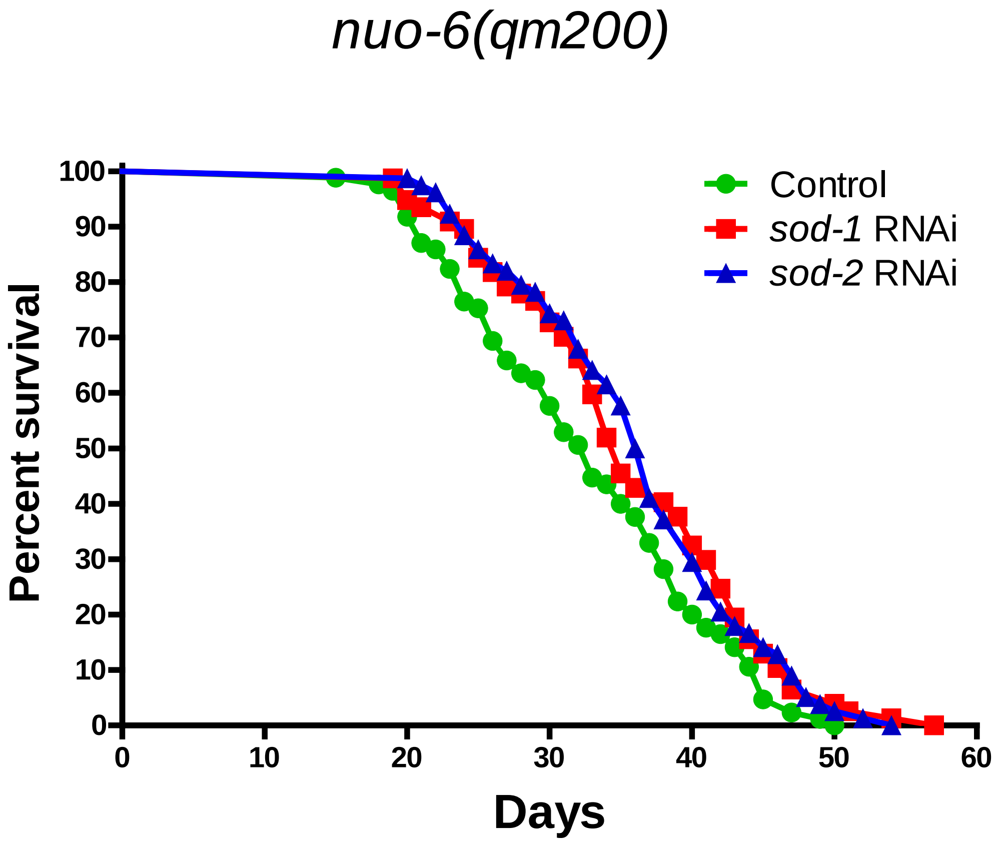

Knocking down *sod-1* (red) or *sod-2* (blue) does not shorten the long lifespan of *nuo-6(qm200)* mutant. In fact silencing these two genes slightly increases the lifespan of *nuo-6* mutants. Mean lifespan of control (empty vector) is 33 days (green), mean lifespan after *sod-1* RNAi treatment is 35 days and mean lifespan after *sod-2* RNAi treatment is 36.5 days, $p<0.05$ for both RNAi experiments compare to control, analyzed by curve comparison using the log-rank test.
