# Supplementary material for: A Mitochondrial Superoxide Signal Triggers Increased Longevity in Caenorhabditis elegans
Source: PLoS Biol. 2010 Dec 7;8(12):e1000556. doi: 10.1371/journal.pbio.1000556 (PMC2998438; doi:10.1371/journal.pbio.1000556)
Supplement: Table S1 — Individual aging experiments and statistics. (0.14 MB PDF) [file pbio.1000556.s006.pdf]

Supporting Table S1: Individual aging experiments and statistics

Genotype	Treatment	Mean lifespan (days)	Maximum lifespan (days)	p-values vs. control*	Mean lifespan change (days)
**Wild type (N2)**	Control[#]	19.1 ± 3.4 (n =50)	28		
	0.1 mM paraquat (PQ)	31.0 ± 4.7 (n =50)	40	P<0.0001	+10.9
**Wild type (N2)**	Control	15.0 ± 3.1 (n =50)	22		
	0.05 mM PQ	22.0 ± 2.7 (n =46)	30	P<0.0001	+7.0
	0.1 mM PQ	28.9 ± 6.8 (n =50)	40	P<0.0001	+13.9
	0.2 mM PQ	21.1 ± 4.7 (n =45)	27	P<0.0001	+6.1
**Wild type (N2)**	Control	17.8 ± 3.4 (n =50)	25		
	0.1 mM PQ entire life	28.7 ± 6.5 (n =50)	43	P<0.0001	+10.9
	0.1 mM PQ larval dev.	22.9 ± 5.2 (n =50)	32	P<0.0001	+5.1
	0.1 mM PQ adulthood	27.1 ± 6.9 (n =50)	40	P<0.0001	+9.3
**Wild type (N2)**	Empty vector (HT115) to OP50**	20.9 ± 3.8 (n =50)	27		
		19.6 ± 3.3 (n =50)	26		
	Empty vector (HT115) to 0.1 mM PQ (OP50)**	23.5 ± 4.8 (n =50)	30		+3.4
		22.7 ± 4.2 (n =50)	31	P<0.0001	+2.6
**Wild type (N2)**	*nuo-6* RNAi to OP50**	24.4 ± 4.4 (n =50)	32		
		22.2 ± 4.6 (n =50)	28		
	nuo-6 RNAi to 0.1 mM PQ (OP50)**	31.7 ± 3.5 (n =50)	41		+8.1
		28.6 ± 3.3 (n =50)	33	P<0.0001	+6.3
**Wild type (N2)**	*isp-1* RNAi to OP50**	22.4 ± 5.4 (n =50)	34		
		23.5 ± 6.2 (n =50)	32		
	i*sp-1* RNAi to 0.1 mM PQ (OP50)**	25.5 ± 4.8 (n =50)	36		+2.5
		24.5 ± 5.1 (n =50)	35		+1.5
**Wild type (N2)**	Dead bacteria	23.0 ± 4.2 (n =50)	30		

Supporting Table S1

	Dead bact. + 0.1 mM PQ	30.4 ± 3.2 (n =50)	37	P<0.0001	+16.6
clk-1(qm30)	Dead bacteria	25.9 ± 6.9 (n =50)	40		
	Dead bact + 0.1 mM PQ	37.3 ± 3.4 (n =50)	44	P<0.0001	+11.4
isp-1(qm150)	Dead bacteria	36.2 ± 7.1 (n =29)	55		
	Dead bact. + 0.1 mM PQ	36.8 ± 6.5 (n =30)	49	P=0.8489	+0.6
nuo-6(qm200)	Dead bacteria	38.5 ± 5.8 (n =30)	49		
	Dead bact. + 0.1 mM PQ	38.0 ± 5.7 (n =27)	49	P=0.9777	-0.5
daf-2(e1370)	Dead bacteria	41.7 ± 9.4 (n =50)	59		
	Dead bact.+ 0.1 mM PQ	60.9 ± 9.2 (n =20)	83	P<0.0001	+19.2
daf-16(m26)	Dead bacteria	23.1 ± 2.9 (n =50)	27		
	Dead bact. + 0.1 mM PQ	26.0 ± 2.8 (n =50)	31	P<0.0001	+2.9
**Wild type (N2)**	Control	19.3 ± 3.4 (n =50)	24		
	Benzyl viologen	26.9 ± 3.4 (n =50)	29	P<0.0001	+7.6
	Benzyl viologen	26.4 ± 3.8 (n =50)	30		+7.1
**Wild type (N2)**	Control	22.0 ± 3.1 (n =50)	28		
	Control	18.7 ± 4.0 (n =50)	27		
	Control	21.0 ± 4.4 (n =50)	29		
	0.1 mM PQ	27.8 ± 3.4 (n =50)	35	P<0.0001	+7.2
	0.1 mM PQ	29.4 ± 5.7 (n =50)	30		+8.8
	0.1 mM PQ	28.6 ± 5.0 (n =50)	39		+8.0
**Wild type (N2)**	Control	18.5 ± 3.0 (n =50)	24	P=0.0127 (P=0.0010)[+]	
clk-1(qm30)	Control	20.9 ± 4.1 (n =50)	30		
	Control	20.3 ± 3.1 (n =50)	29		
	Control	21.1 ± 4.4 (n =50)	33		
	0.1 mM PQ	34.3 ± 5.2 (n =50)	43	P<0.0001	+13.5
	0.1 mM PQ	34.7 ± 5.6 (n =50)	45		+13.9
	0.1 mM PQ	40.4 ± 7.7 (n =50)	58		+19.6

Supporting Table S1

	0.1 mM PQ	38.0 ± 9.0 (n =50)	53		+17.2
daf-2(e1370)	Control	45.9 ± 8.8 (n =50)	67		
	Control	44.1 ± 6.0 (n =50)	55		
	Control	41.4 ± 11.0 (n =50)	65		
	0.1 mM PQ	42.9 ± 11.0 (n =50)	54	P<0.0001	-0.8
	0.1 mM PQ	53.7 ± 10.8 (n =50)	76		+9.9
	0.1 mM PQ	51.1 ± 8.5 (n =50)	70		+7.3
daf-2(e1370); nuo-6(qm200)	Control	47.3 ± 15.9 (n =45)	81		+5.5
	Control	48.6 ± 16.3 (n =50)	76	P<0.0001	+6.8
isp-1(qm150)	Control	33.6 ± 7.9 (n =50)	53		
	Control	33.5 ± 7.8 (n =50)	50		
	Control	34.3 ± 7.9 (n =50)	50		
	0.1 mM PQ	34.2 ± 7.3 (n =50)	50	P=0.0961	+0.4
	0.1 mM PQ	33.3 ± 6.5 (n =50)	50		-0.5
	0.1 mM PQ	37.3 ± 5.0 (n =50)	47		+3.5
	0.1 mM PQ	30.6 ± 8.3 (n =50)	51		-3.2
nuo-6(qm200)	Control	35.9 ± 8.2 (n =50)	49		
	Control	30.7 ± 7.6 (n =50)	50		
	Control	32.4 ± 7.2 (n =50)	46		
	Control	33.5 ± 7.5 (n =50)	48		
	Control	34.2 ± 7.1 (n =50)	47		+1.1
	0.1 mM PQ	38.4 ± 6.4 (n =50)	52	P=0.0125	+5.3
	0.1 mM PQ	34.6 ± 9.9 (n =50)	54		+1.5
	0.1 mM PQ	34.5 ± 7.2 (n =50)	46		+1.4
	0.1 mM PQ	35.3 ± 8.5 (n =50)	53		+2.2
	0.1 mM PQ	34.6 ± 9.9 (n =50)	54		+1.5
sod-2(ok1030)	Control	23.1 ± 4.6 (n =50)	38		

Supporting Table S1

	Control	21.3 ± 4.6 (n =50)	33		
	0.1 mM PQ	26.2 ± 4.0 (n =50)	36	P<0.0001	+4.0
	0.1 mM PQ	31.4 ± 4.1 (n =50)	39		+9.2
daf-16(m26)	Control	19.1 ± 2.9 (n =50)	23		
	Control	16.9 ± 2.1 (n =50)	21		
	Control	16.5 ± 8.5 (n =50)	20		
	Control	23.1 ± 2.9 (n =50)	27		
	0.1 mM PQ	22.0 ± 2.8 (n =50)	27	P<0.0001	+3.1
	0.1 mM PQ	22.5 ± 3.9 (n =50)	28		+3.6
	0.1 mM PQ	22.1 ± 8.5 (n =50)	28		+3.2
	0.1 mM PQ	26.0 ± 2.8 (n =50)	31		+7.1
aak-2(ok524)	Control	17.6 ± 2.5 (n =50)	22		
	Control	17.0 ± 2.4 (n =50)	22		
	0.1 mM PQ	21.1 ± 3.3 (n =50)	29	P<0.0001	+3.8
	0.1 mM PQ	22.8 ± 5.2 (n =50)	30		+5.5
hsf-1(y441)	Control	15.9 ± 3.1 (n =50)	22		
	Control	13.9 ± 2.7 (n =50)	20		
	0.1 mM PQ	25.6 ± 6.1 (n =50)	37	P<0.0001	+10.7
	0.1 mM PQ	21.3 ± 4.2 (n =50)	32		+6.4
hif-1(ia4)	Control	23.4 ± 4.1 (n =50)	30		
	0.1 mM PQ	28.7 ± 6.8 (n =50)	41	P<0.0001	+5.3
**Wild type (N2)**	Control	19.2 ± 2.5 (n =50)	24		
	Control	18.5 ± 2.8 (n =50)	24		
	0.1 mM PQ on adult	25.2 ± 3.1 (n =50)	29	P<0.0001	+6.3
	0.1 mM PQ on adult	25.3 ± 3.7 (n =50)	30		+6.4
wwp-1(ok1102)	Control	22.3 ± 3.0 (n =50)	28		
	Control	21.5 ± 2.9 (n =50)	28		

Supporting Table S1

	0.1 mM PQ on adult	30.0 ± 5.6 (n =50)	37	P<0.0001	+8.1
	0.1 mM PQ on adult	28.1 ± 4.6 (n =50)	34		+6.2
skn-1(zn67)	Control	17.6 ± 2.3 (n =50)	22		
	0.1 mM PQ on adult	22.8 ± 2.8 (n =50)	25	P<0.0001	+5.2
nuo-6(qm200)	Empty vector (HT115)	32.0 ± 9.0 (n =40)	49		
	Empty vector (HT115)	32.4 ± 8.6 (n =40)	48		
	sod-1 RNAi	34.4 ± 7.0 (n =40)	44	P<0.0001	+2.2
	sod-1 RNAi	37.3 ± 9.8 (n =40)	56		+5.1
	sod-2 RNAi	36.0 ± 8.3 (n =40)	51	P<0.0001	+3.6
	sod-2 RNAi	36.4 ± 7.8 (n =40)	53		+4.2
**Wild type (N2)**	Control	22.1 ± 3.7 (n =50)	28		
	Control	19.4 ± 3.4 (n =50)	27		
	1 mM NAC	20.2 ± 3.4 (n =50)	27		-0.6
	10 mM NAC	19.6 ± 2.6 (n =50)	26	P=0.1655	-1.2
	10 mM NAC	20.8 ± 3.6 (n =50)	30		-0.0
**Wild type (N2)**	Control	20.3 ± 3.0 (n =50)	27		
	15 mM NAC	21.6 ± 4.2 (n =50)	31		+1.3
	1 mM Vit C	21.0 ± 3.0 (n =50)	28		+0.7
**Wild type (N2)**	Control	17.6 ± 2.5 (n =50)	22		
	8 mM NAC	16.6 ± 3.2 (n =37)	21		-1.0
	10 mM NAC	16.5 ± 2.5 (n =42)	20		-1.1
nuo-6(qm200)	Control	33.4 ± 7.6 (n =50)	50		
	Control	33.4 ± 9.8 (n =50)	54		
	1 mM NAC	31.2 ± 9.0 (n =50)	45		-2.2
	10 mM NAC	21.1 ± 2.2 (n =38)	25	P<0.0001	-12.3
	10 mM NAC	20.1 ± 3.6 (n =35)	28		-13.3
nuo-6(qm200)	Control	32.8 ± 6.9 (n =50)	47		

Supporting Table S1

	0.2 mM PQ	27.8 ± 7.2 (n =27)	39		-4.0
	1 mM Vit C	25.8 ± 6.2 (n =50)	37	P<0.001	-6.0
	1 mM Vit C	27.0 ± 7.5 (n =50)	41	P<0.001	-5.2
clk-1(qm30)	Control	22.1 ± 3.0 (n =46)	33		
	Control	22.8 ± 4.3 (n =46)	33		
	10 mM NAC	22.2 ± 3.3 (n =50)	30	P=0.4247	-0.3
	10 mM NAC	23.7 ± 4.4 (n =50)	30		+1.2
clk-1(qm30)	Control	22.4 ± 4.4 (n =50)	34		
	Control	22.3 ± 5.2 (n =50)	37		
	15 mM NAC.	22.2 ± 2.0 (n =50)	27[$]		-0.1
	15 mM NAC	22.2 ± 2.0 (n =50)	27		-0.1
isp-1(qm150)	Control	34.1 ± 8.2 (n =50)	47		
	Control	33.9 ± 7.9 (n =50)	53		
	10 mM NAC	21.6 ± 2.9 (n =50)	28	P<0.0001	-11.9
	10 mM NAC	28.5 ± 5.6 (n =50)	38		-5.0
	10 mM NAC	25.8 ± 4.6 (n=50)	35		-7.7
	10 mM NAC	26.6 ± 6.2 (n =50)	37		-6.9
isp-1(qm150)	Control	32.2 ± 8.2 (n =50)	48		
	1 mM NAC	30.7 ± 9.0 (n =50)	48		
	1 mM NAC.	32.0 ± 9.6 (n =50)	47		
	8 mM NAC	27.1 ± 4.3 (n =50)	33		-5.1
	10 mM NAC	21.0 ± 2.6 (n =50)	27	P<0.001	-9.5
	0.2 mM PQ	27.8 ± 7.5 (n =50)	43	P<0.001	-2.7
	1 mM Vit C	25.1 ± 4.8 (n =50)	34	P<0.001	-5.4
	1 mM Vit C	24.2 ± 5.0 (n =50)	39	P<0.001	-6.3
sod-2(ok1030)	Control	24.7 ± 7.7 (n =50)	41		

Supporting Table S1

	Control	23.0 ± 6.0 (n =50)	41		
	10 mM NAC	20.8 ± 3.2 (n =50)	29	P<0.0001	-3.1
	10 mM NAC	19.6 ± 3.9 (n =50)	30		-4.3
daf-2(e1370)	Control	40.1 ± 11.6 (n =50)	64		
	Control	43.1 ± 9.7 (n =50)	67		
	10 mM NAC	33.5 ± 9.6 (n =50)	54	P<0.0001	-8.3
	10 mM NAC	39.2 ± 9.3 (n =50)	54		-2.6

*When experiments have been carried out more than once the p-values provided are for comparisons between pooled datasets for each condition.

[#]When the use of the RNAi strain HT115 is not specified it means that experiments were carried out with OP50.

**In these experiments worms were transferred from HT115 bacteria (with empty vector or a particular clone) to OP50 at the young adult stage, at which time they are treated or not with PQ. This was because HT115 bacteria are too sensitive to PQ to remain a good food source for the worms.

[+]For this particular experiment, because of the relatively moderate increase in *clk-1* lifespan, we have added the lifespan of wild type controls that were carried out at the same time. In brackets we also provide the statistical significance of the difference in lifespan between the *clk-1* mutants in this experiments and the pool of the wild type from all experiments.
